# Recovery of organ-specific tissue oxygen delivery at restrictive transfusion thresholds after fluid treatment in ovine haemorrhagic shock

**DOI:** 10.1186/s40635-022-00439-6

**Published:** 2022-04-04

**Authors:** Wayne B. Dyer, Gabriela Simonova, Sara Chiaretti, Mahe Bouquet, Rebecca Wellburn, Silver Heinsar, Carmen Ainola, Karin Wildi, Kei Sato, Samantha Livingstone, Jacky Y. Suen, David O. Irving, John-Paul Tung, Gianluigi li Bassi, John F. Fraser

**Affiliations:** 1 Australian Red Cross Lifeblood, Sydney, Australia; 2 Australian Red Cross Lifeblood, Brisbane, Australia; 3 Critical Care Research Group, The Prince Charles Hospital, Brisbane, Australia; 4 Faculty of Medicine, The University of Queensland, Brisbane, Australia; 5 Faculty of Health, Queensland University of Technology, Brisbane, Australia; 6 Cardiovascular Research Institute, Basel, Switzerland; 7 Faculty of Health, University of Technology, Sydney, Australia; 8 Medical Engineering Research Facility, Queensland University of Technology, Brisbane, Australia; 9 Institut d’Investigacions Biomèdiques August Pi i Sunyer, Barcelona, Spain

**Keywords:** Haemorrhagic shock, Patient blood management, Tissue oxygen delivery, Oxygen debt, Microcirculation, Haemodilution, Transfusion thresholds

## Abstract

**Background:**

Fluid resuscitation is the standard treatment to restore circulating blood volume and pressure after massive haemorrhage and shock. Packed red blood cells (PRBC) are transfused to restore haemoglobin levels. Restoration of microcirculatory flow and tissue oxygen delivery is critical for organ and patient survival, but these parameters are infrequently measured. Patient Blood Management is a multidisciplinary approach to manage and conserve a patient’s own blood, directing treatment options based on broad clinical assessment beyond haemoglobin alone, for which tissue perfusion and oxygenation could be useful. Our aim was to assess utility of non-invasive tissue-specific measures to compare PRBC transfusion with novel crystalloid treatments for haemorrhagic shock.

**Methods:**

A model of severe haemorrhagic shock was developed in an intensive care setting, with controlled haemorrhage in sheep according to pressure (mean arterial pressure 30–40 mmHg) and oxygen debt (lactate > 4 mM) targets. We compared PRBC transfusion to fluid resuscitation with either PlasmaLyte or a novel crystalloid. Efficacy was assessed according to recovery of haemodynamic parameters and non-invasive measures of sublingual microcirculatory flow, regional tissue oxygen saturation, repayment of oxygen debt (arterial lactate), and a panel of inflammatory and organ function markers. Invasive measurements of tissue perfusion, oxygen tension and lactate levels were performed in brain, kidney, liver, and skeletal muscle. Outcomes were assessed during 4 h treatment and post-mortem, and analysed by one- and two-way ANOVA.

**Results:**

Each treatment restored haemodynamic and tissue oxygen delivery parameters equivalently (*p* > 0.05), despite haemodilution after crystalloid infusion to haemoglobin concentrations below 70 g/L (*p* < 0.001). Recovery of vital organ-specific perfusion and oxygen tension commenced shortly before non-invasive measures improved. Lactate declined in all tissues and correlated with arterial lactate levels (*p* < 0.0001). The novel crystalloid supported rapid peripheral vasodilation (*p* = 0.014) and tended to achieve tissue oxygen delivery targets earlier. PRBC supported earlier renal oxygen delivery (*p* = 0.012) but delayed peripheral perfusion (*p* = 0.034).

**Conclusions:**

Crystalloids supported vital organ oxygen delivery after massive haemorrhage, despite haemodilution to < 70 g/L, confirming that restrictive transfusion thresholds are appropriate to support oxygen delivery. Non-invasive tissue perfusion and oximetry technologies merit further clinical appraisal to guide treatment for massive haemorrhage in the context of Patient Blood Management.

**Supplementary Information:**

The online version contains supplementary material available at 10.1186/s40635-022-00439-6.

## Introduction

Uncontrolled haemorrhage and shock results in over 1.9 million deaths worldwide annually, with trauma, surgical bleeding and post-partum haemorrhage the primary causes [1]. The first priority when treating acute trauma haemorrhage is mechanical or haemostatic cessation of major bleeding, followed closely by sufficient fluids to increase oxygen delivery to vital organs, in the context of permissive hypotension, until major sources of bleeding have ceased [2–4]. The same principle applies in acute non-trauma and surgical haemorrhage [5]. Early use of tranexamic acid and clotting factor concentrates can significantly reduce persistent haemorrhage and improve survival [6, 7], while pre-hospital packed red blood cell (PRBC) transfusion alone, which is often used in such circumstances, may not improve overall survival [8]. Early haemostatic resuscitation with balanced blood components [9], and more recently whole blood [10], are increasingly used in the pre-hospital setting to increase blood volume and stabilise haemodynamic parameters [2], and have improved short-term survival into hospital [11].

Upon cessation of major haemorrhage and subsequent volume replacement, current guidelines recommend PRBC transfusion only if haemoglobin is below the restrictive threshold of 70 g/L [12–14]. To reduce risks from exposure to allogeneic blood products [15, 16], crystalloids or colloid-based solutions may be preferred to PRBC in controlled haemorrhage scenarios, even if haemoglobin levels are reduced below the restrictive transfusion threshold [2, 17]. The application of Patient Blood Management includes conservation of a patient’s own blood, tolerance of anaemia, and optimised regeneration of lost blood cells. In this context, the decision to transfuse or use another treatment should be based on broad clinical assessment, not just haemoglobin levels [12–14]. For example, microvascular flow and tissue oxygenation are critical for organ function and survival, and, therefore, could be used more widely to inform treatment decisions [2]. Furthermore, transfusion to haemoglobin levels alone may not necessarily improve tissue oxygen delivery unless microvascular perfusion was deficient before treatment [18]. Since effective oxygen exchange at the tissue level requires functional capillary density [19], evaluation of microcirculatory blood flow as a treatment decision tool is compelling [2]. In addition, second-generation Near Infra-Red Spectroscopy (NIRS) platforms offer improved reliability in monitoring oxyhaemoglobin saturation in cerebral and peripheral tissues [20]. Elevated blood lactate is a reliable indicator of oxygen debt status to monitor treatment efficacy [21]. With further technological advances in these platforms, their reliability in predicting critical tissue oxygen delivery in vital organs warrants further investigation.

Optimal treatment for massive haemorrhage remains a contested issue, but an individualised approach in the context of Patient Blood Management is strongly supported [2, 22–24]. PlasmaLyte is a balanced crystalloid for treating haemorrhage and critical illness [25]. An experimental balanced crystalloid under development, described as an isotonic crystalloid aqueous solution (ICAS) containing nitrate and nitrite ions, metals and metalloids [26], restored microvascular flow, tissue oxygen delivery and repayment of oxygen debt in a porcine survival model of severe haemorrhagic shock [27]. We compared tissue-specific outcomes between PRBC transfusion and these balanced crystalloids in the controlled haemorrhage setting using the ovine haemorrhagic shock model we described previously [28]. We assessed reliability of non-invasive measures of tissue oxygen delivery to predict organ-specific capillary flow, oxygen delivery and metabolic recovery.

## Methods

### Animals and ethics

The Queensland University of Technology Animal Ethics Committee approved this study (approval #1800000493). We conducted 27 experiments with non-pregnant Dorset-cross ewes, < 3 years. The study was designed according to ARRIVE guidelines, and experiments were conducted according to the *Australian Code for the Care and Use of Animals for Scientific Purposes* [29].

### Resuscitation fluids and randomisation

Sheep blood donor panels were cross-matched with experimental animals. Cross-match reactive sheep were randomised to receive PlasmaLyte (Baxter Healthcare, QLD, Australia), or the investigational crystalloid ICAS; a prototype formulation (sterile isotonic sea water) was purchased from Laboratories Quinton, (Cox, Alicante, Spain) [27]. Cross-match negative sheep were assigned to PRBC transfusion, or randomised to crystalloids. Four PRBC units were produced for each transfusion experiment 2 weeks before use, according to validated protocols replicating production and storage of human PRBC [30].

### Surgical instrumentation and experimental timeline

**Fig. 1:**
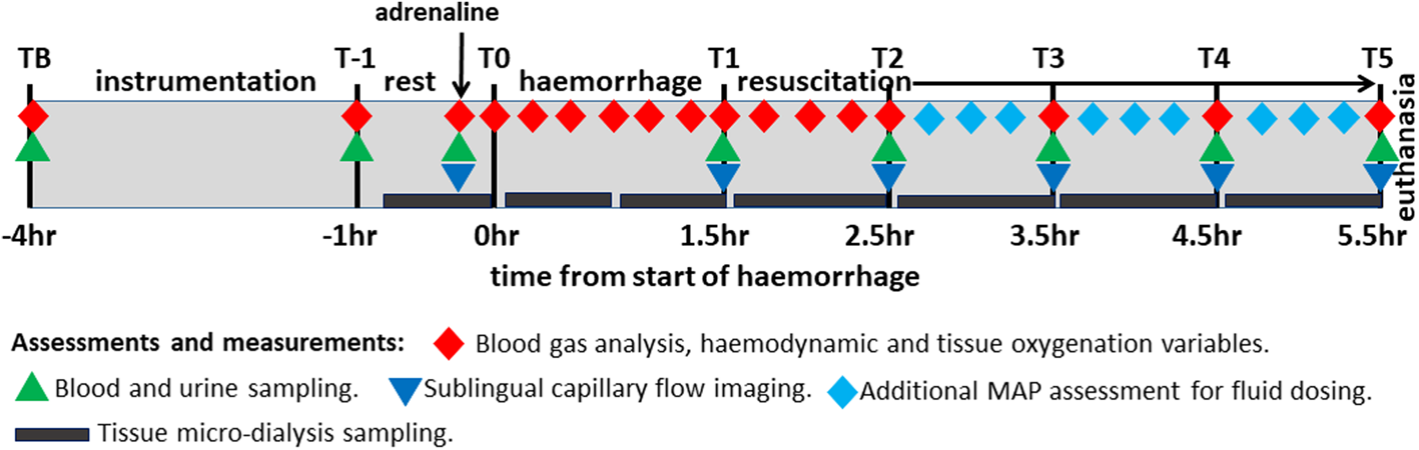
Experimental timeline and summary of assessments

Animals were induced and remained under general anaesthesia and mechanical ventilation, surgically instrumented in the right-side up position, and comprehensively monitored according to standard clinical practice as described in detail elsewhere [28, 31]. Invasive measures of microvascular flow and oxygen tension (Oxford Optronix, UK) and micro-dialysis (M Dialysis AB, Sweden) were calibrated and used according to the manufacturer’s instructions. To reverse the effects of anaesthesia-associated splenic relaxation on haematocrit to determine total haemoglobin at experimental baseline [32, 33], adrenaline (0.05–0.15 mcg/kg/min) was given briefly to constrict the spleen [34, 35]. The experimental timeline and summary of sampling and assessments is shown in Fig. 1.

### Haemorrhage and shock protocol

Haemorrhagic shock was induced as described [28], drawing 40–60% total blood volume (%TBV) of venous blood until mean arterial pressure (MAP) < 40 mmHg and oxygen debt defined by lactate > 4 mM was achieved. Haemorrhage was paused when MAP < 30 mmHg and/or heart rate (HR) > 200 bpm. Haemorrhage proceeded for 90 min or until development of shock targets (maximum 120 min).

### Resuscitation protocols

In sheep resuscitated with PlasmaLyte or ICAS, fluids were dosed to achieve MAP > 65 mmHg [28]. An initial 1000 mL bolus was given within 10 min, and fluid requirements were assessed every 15 min. If MAP > 65 mmHg, fluids were tapered to 20 mL/kg/h, then 10 mL/kg/h, and ultimately ceased. If MAP subsequently decreased, fluid dosing increased accordingly, and vasoactive drugs were given if MAP remained < 50 mmHg [28].

In sheep resuscitated with PRBC and Hartmann’s, fluid infusion commenced at 20 mL/kg/h and the first PRBC unit was transfused within 10 min via a separate jugular sheath. The second unit was transfused within 15–20 min. Remaining units were transfused if MAP < 65 mmHg; the total number did not exceed the number of whole blood units lost to haemorrhage. Thereafter, Hartmann’s was continued as per the crystalloid protocol above, with vasopressor drug support as required.

All sheep were monitored for 4 h following commencement of resuscitation. After this, sheep were euthanised as described [28], and post-mortem assessments completed.

### Primary and secondary haemodynamic and oxygen delivery outcomes

The primary composite haemodynamic outcome was time to achieve MAP ≥ 65 mmHg and cardiac index (CI) ≥ 2.5 L/min/m^2^. Secondary haemodynamic targets included HR < 120 bpm, systemic vascular resistance index (SVRI) < 2390 dynes*s/cm^5^/m^2^, and PaO_2_/FiO_2_ ratio > 300. The haemoglobin range for sheep (73–116 g/L) established at our facility [36] is lower than commonly reported (90–150 g/L).

The primary composite tissue oxygen delivery outcome was time to achieve peripheral muscle regional tissue oxygen saturation (StO_2_) > 50% and arterial lactate < 2 mM. Secondary oxygen delivery targets included mixed venous saturation (SvO_2_) > 60%, brain StO_2_ > 60%, and base excess > − 2 mM.

### Organ-specific outcomes

Investigational organ-specific outcomes included > 75% recovery-to-baseline for tissue oxygen tension (PtO_2_) and microvascular flow, reduction of tissue lactate to within one standard deviation of baseline, and a lactate/pyruvate ratio < 30. Sublingual microvascular perfusion, measured and calculated by Cytocam software (Braedius Medical, The Netherlands), recovered if Proportion Perfused Vessels (PPV) > 75% baseline.

### Post-mortem and laboratory assessments

Mitochondrial function in right ventricle and renal cortex tissue was performed by high resolution respirometry (O2k-Oxygraph; Oroboros Instruments, Innsbruck, Austria); see method in Additional file 1. Plasma levels of inflammatory cytokines, hyaluronan and cardiac troponin-I were measured by sheep-specific ELISA as described [28, 37], or pig-hsCTn-I ELISA (Life Diagnostics). Full blood counts were performed on the Mindray BC-5000 Vet analyser, and viscoelastic tests by ROTEM [28]. Serum biochemistry and urinalysis was performed by QML-Vetnostics.

### Statistical analyses

Statistical analyses were performed in Prism (version 8). All data were tested for normality (Kolmogorov–Smirnov) and subsequent tests chosen accordingly. Baseline measurements are presented as mean and SD, and tested by one-way ANOVA or Kruskal–Wallis, with Tukey correction. Time-based observations between groups were presented as mean or geometric mean with 95% confident intervals, and analysed by mixed-effects models (repeated measures ANOVA) with Tukey post-hoc correction. Outliers were excluded by ROUT. Time-to-treat analysis of primary outcomes was performed by the Mantel–Cox log-rank test. End-point outcomes were compared by Mann Whitney or unpaired *t* tests. Clinical measures of tissue oxygen delivery and debt were correlated with organ-specific measures by Spearman or Pearson tests. *p* values (including recommended post-tests and correction where appropriate) < 0.05 were considered significant. All statements of similarity between groups imply non-significance (*p* values > 0.05).

## Results

We conducted 27 experiments with non-pregnant Dorset-cross ewes, < 3 years, of which 24 are reported here (eight sheep per treatment group). Two were excluded after pre-existing pulmonary conditions were evident, and one excluded after an adverse response to haemorrhage.

### Baseline characteristics and treatment variables

Distribution of baseline characteristics and treatment variables suggested randomisation and treatment allocation was effective (Table 1). Primary and secondary clinical measures were similar at baseline, except mild tachycardia in the PlasmaLyte group.

**Table 1:** Baseline characteristics and treatment variables, and baseline primary outcome measures

	Baseline^a^	Treatment groups: mean (SD)			Group comparison^c^
	Range	1-PlasmaLyte	2-ICAS	3-PRBC	(*p* value)
Animals (*n* =)		8	8	8	–
Weight (kg)		51.5 (5.2)	50.9 (7.7)	52.3 (6.3)	0.75
Haemorrhage time (min)		68 (15)	74 (16)	78 (14)	0.49
Total haemorrhage (mL)^b^		1427 (177)	1630 (358)	1694 (328)	0.067
Haemorrhage (%TBV)		41.4 (5.2)	46.8 (6.2)	48.5 (9.5)	0.145
Total resus volume (L)		3.64 (1.63)	4.07 (0.54)	3.16 (0.89)	0.28
Resus/haem volume ratio		2.5 (0.9)	2.6 (0.4)	1.9 (0.5)	0.117
Resus rate (mL/kg/h)		17.5 (6.9)	21.0 (3.3)	15.2 (4.0)	0.087
NorAd use per group		4/8	4/8	3/8	–
NorAd, total dose (mcg/kg)		0.0318 (0.0149)	0.0228 (0.0134)	0.0100 (0.0009)	0.87
Other vasopressor use		Dopamine: 1/8	Metaraminol: 1/8	0/8	–
MAP (mmHg)	66–95	84 (9)	84 (8)	92 (23)	0.50
Cardiac index (L/min/m^2^)	1.9–6.5	4.2 (1.6)	3.1 (0.7)	3.8 (0.6)	0.144
Heart rate (beats/min)	58–128	111 (30)	94 (20)	86 (18)	0.048
SVRI (dynes*s/cm^5^/m^2^)	800–3200	1645 (789)	2069 (436)	1875 (403)	0.137
PaO_2_/FiO_2_ ratio	229–543	338 (77)	345 (95)	425 (90)	0.112
Hb, post-adrenaline (g/L)	94–14.8	112 (14)	123 (16)	125 (18)	0.26
SvO_2_ (%)	57–85	71 (9)	74 (8)	72 (7)	0.76
Brain StO_2_ (%)	51–88	76 (10)	72 (8)	67 (8)	0.132
Muscle StO_2_ (%)	56–78	62 (10)	69 (9)	66 (6)	0.27
Arterial lactate (mM)	0.4–1.9	1.7 (2.1)	0.7 (0.2)	1.2 (1.0)	0.192
Base excess (mM)	− 4.7 to + 5.7	1.8 (2.8)	2.3 (2.5)	0.6 (3.3)	0.92

*TBV* total blood volume, *NorAd* noradrenaline, *MAP* mean arterial pressure, *SVRI* systemic vascular resistance index, *Hb* haemoglobin, *StO*_*2*_ regional tissue oxygen saturation, *SvO*_*2*_ mixed venous saturation

^a^Baseline range: 2.5–97.5th percentile

^b^Total haemorrhage included 250 ml iatrogenic surgical and sampling loss

^c^ANOVA or Kruskal–Wallis

### Primary and secondary haemodynamic outcomes

**Fig. 2:**
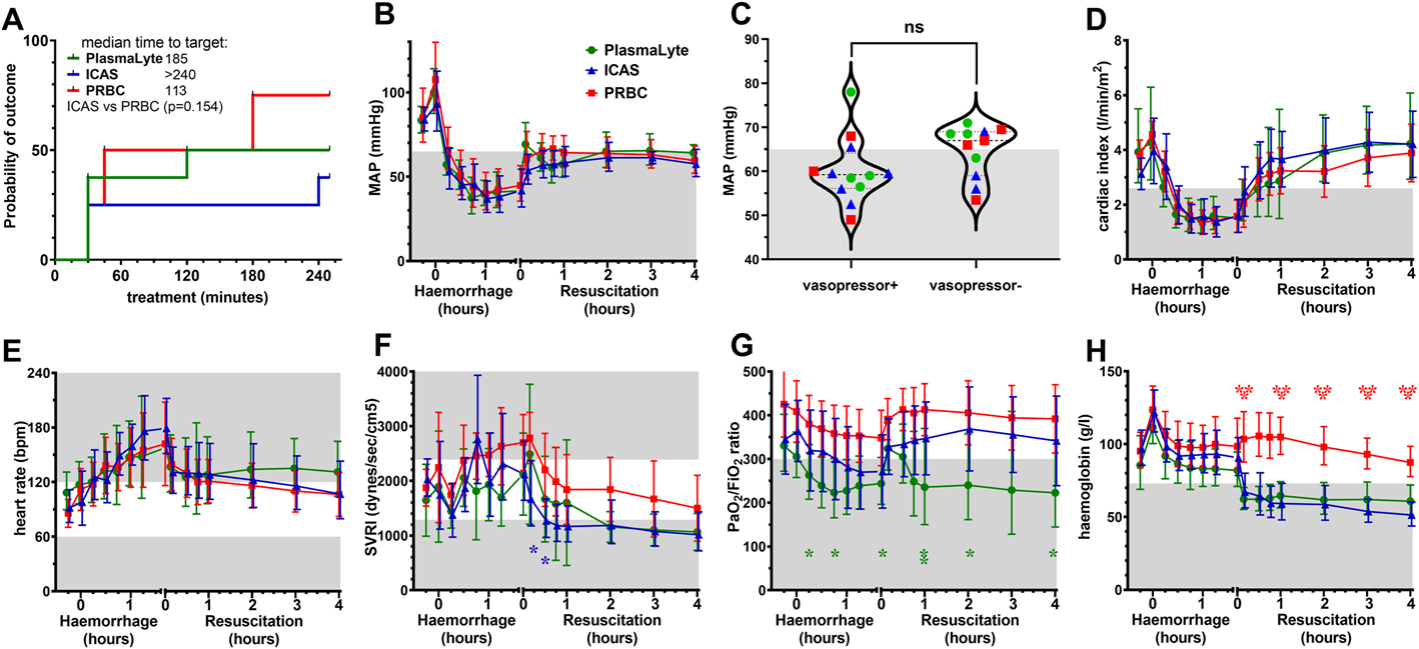
Primary and secondary haemodynamic outcomes. **A** Time to treat analysis of composite haemodynamic target (MAP ≥ 65 mmHg and CI ≥ 2.5 L/min/m^2^). **B** Mean arterial pressure (MAP). **C** Vasopressor use and MAP during the final treatment hour. **D** Cardiac index. **E** Heart rate. **F** Systemic vascular resistance index. **G** *P*/*F* ratio; and (**H**) haemoglobin. Shaded areas represent levels outside the normal range, or treatment targets. Data shown as mean or geometric mean according to normality test, with 95% confidence intervals. Mixed model ANOVA with Tukey correction for multiple comparisons; *p < 0.05, **p < 0.01, ***p < 0.001

There was a non-significant trend toward more animals in the PRBC group achieving the primary composite haemodynamic outcome (Fig. 2A). MAP recovered similarly between groups (Fig. 2B). Vasopressors were given to 12 animals distributed between groups, which increased MAP to similar levels observed in vasopressor-free animals, but most remained below the treatment target (Fig. 2C). Crystalloids tended to increased CI more than PRBC transfusion (Fig. 2D).

Secondary haemodynamic outcomes were also similar between groups, including heart rate (Fig. 2E), whereas SVRI was lower during the first 30 min treatment with ICAS than PRBC (Fig. 2F). PaO_2_/FiO_2_ ratios were similar at baseline, then tended to remain lower in PlasmaLyte-treated animals (Fig. 2G). However, haemoglobin levels diverged significantly after treatment (Fig. 2H). After 30 min crystalloid treatment, haemoglobin was decreased from baseline (118 ± 16 g/L to 65 ± 15 g/; (*p* < 0.0001); equivalent to haemodilution at or below the restrictive transfusion threshold of 70 g/L. After 30 min PRBC transfusion, haemoglobin was also reduced from baseline (125 ± 18 g/L to 107 ± 18 g/L; *p* = 0.0334), but remained above what is considered a liberal transfusion threshold at all times (Fig. 2E).

### Primary and secondary tissue oxygen delivery outcomes

**Fig. 3:**
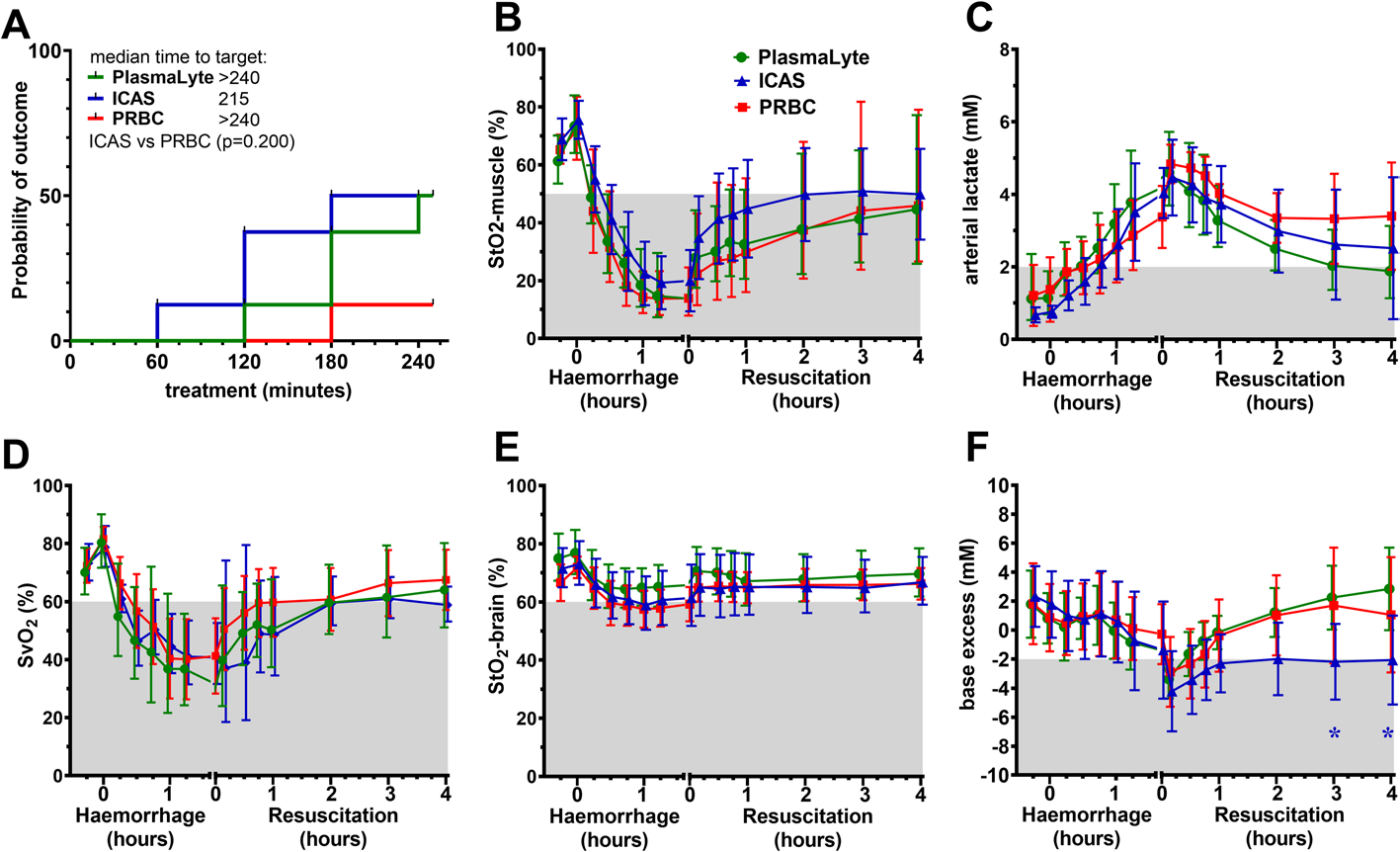
Primary and secondary tissue oxygen delivery outcomes. **A** Time to treat analysis of composite tissue oxygen delivery target (muscle StO_2_ ≥ 50% and arterial lactate ≤ 2 mM). **B** Regional tissue oxygen saturation-muscle. **C** Arterial lactate. **D** Venous oxygen saturation. **E** Regional tissue oxygen saturation-brain. **F** Arterial base excess. Shaded areas represent levels outside the normal range or treatment target. Data shown as mean or geometric mean according to normality test, with 95% confidence intervals. Mixed model ANOVA with Tukey correction for multiple comparisons; *p < 0.05

Overall recovery of tissue oxygen delivery and debt was similar between groups, but the time to achieve the primary composite tissue oxygen delivery outcome (muscle StO_2_ and arterial lactate) tended to be shorter in the ICAS group (Fig. 3A). ICAS treatment tended to support increased muscle StO_2_ (Fig. 3B). Arterial lactate peaked 15 min into resuscitation after the initial fluid bolus flushed acid metabolites from tissues and tended to decline more consistently with crystalloid treatment (Fig. 3C).

The secondary tissue oxygen delivery outcomes were also similar between groups, showing comparable increases in SvO_2_ and brain StO_2_ (Fig. 3D, E). Base consumption after acidic metabolite wash-out was greater after ICAS bolus, and these animals remained in base deficit compared animals that received fluids containing bicarbonate equivalents (PlasmaLyte and Hartmann’s; Fig. 2F).

### Invasive organ-specific oxygen delivery verified clinical measures

**Fig. 4:**
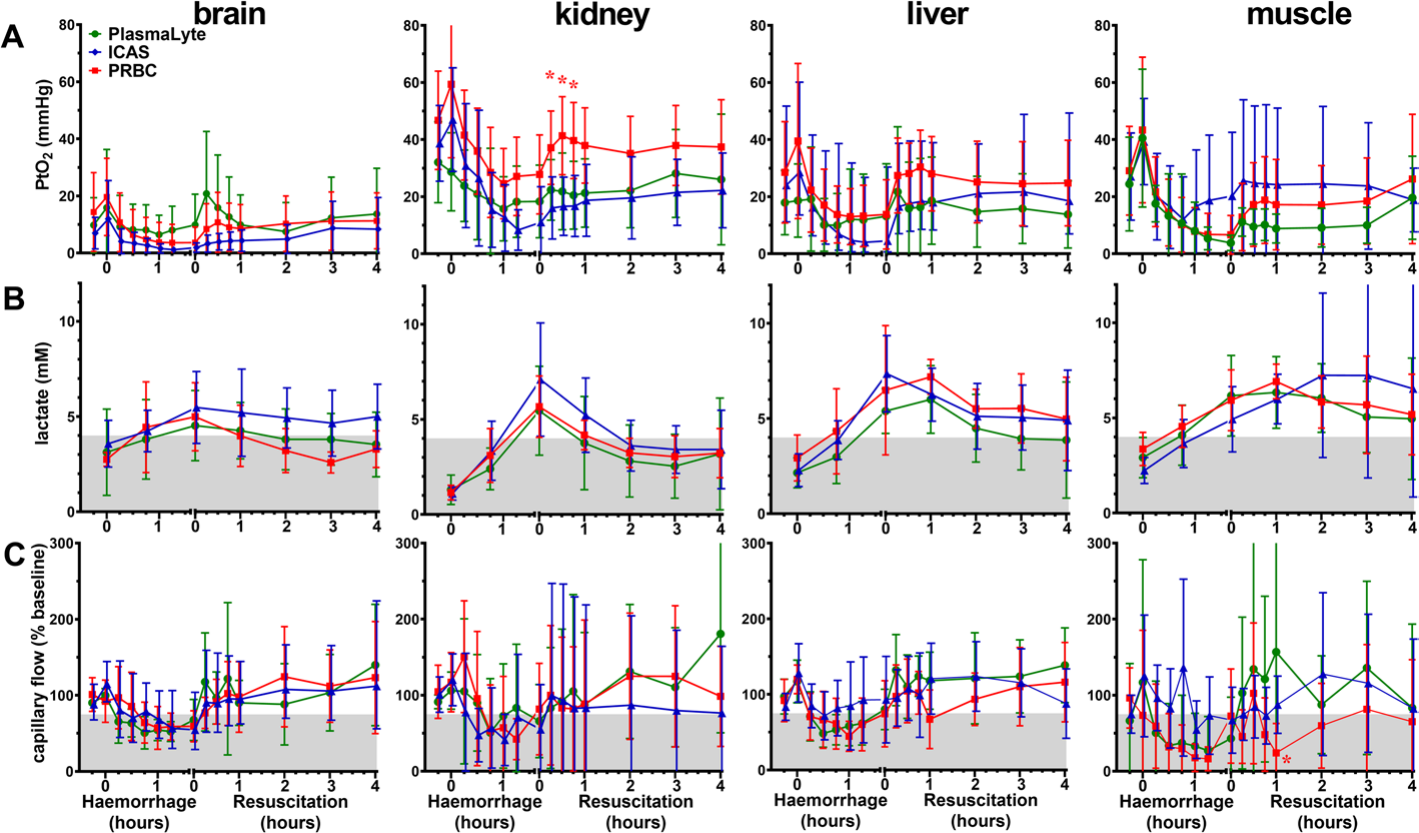
Invasive measures of oxygen tension, lactate concentration and microvascular blood flow in brain, kidney, liver and muscle. **A** Organ-specific oxygen tension (PtO_2_). **B** Organ-specific lactate concentration. **C** Organ-specific microvascular blood flow. Data shown as mean or geometric mean according to normality test, with 95% confidence intervals. Mixed model ANOVA with Tukey correction for multiple comparisons; *p < 0.05

Invasive assessments of organ-specific oxygen tension (PtO_2_), lactate and microvascular flow confirmed recovery of tissue oxygen delivery observed by clinical measures (Fig. 4). Kidney PtO_2_ was higher during the first hour of PRBC than ICAS treatment (*p* = 0.012) but similar thereafter (Fig. 4A). However, kidney and liver PtO_2_ tended to be higher at baseline in PRBC animals. Clearance of lactate from all tissues was equivalent between groups (Fig. 4B). Microvascular flow also partially recovered before resuscitation started, and on average recovered to baseline after 1-h treatment (Fig. 4C), except delayed muscle recovery with PRBC treatment (*p* = 0.034).

**Fig. 5:**
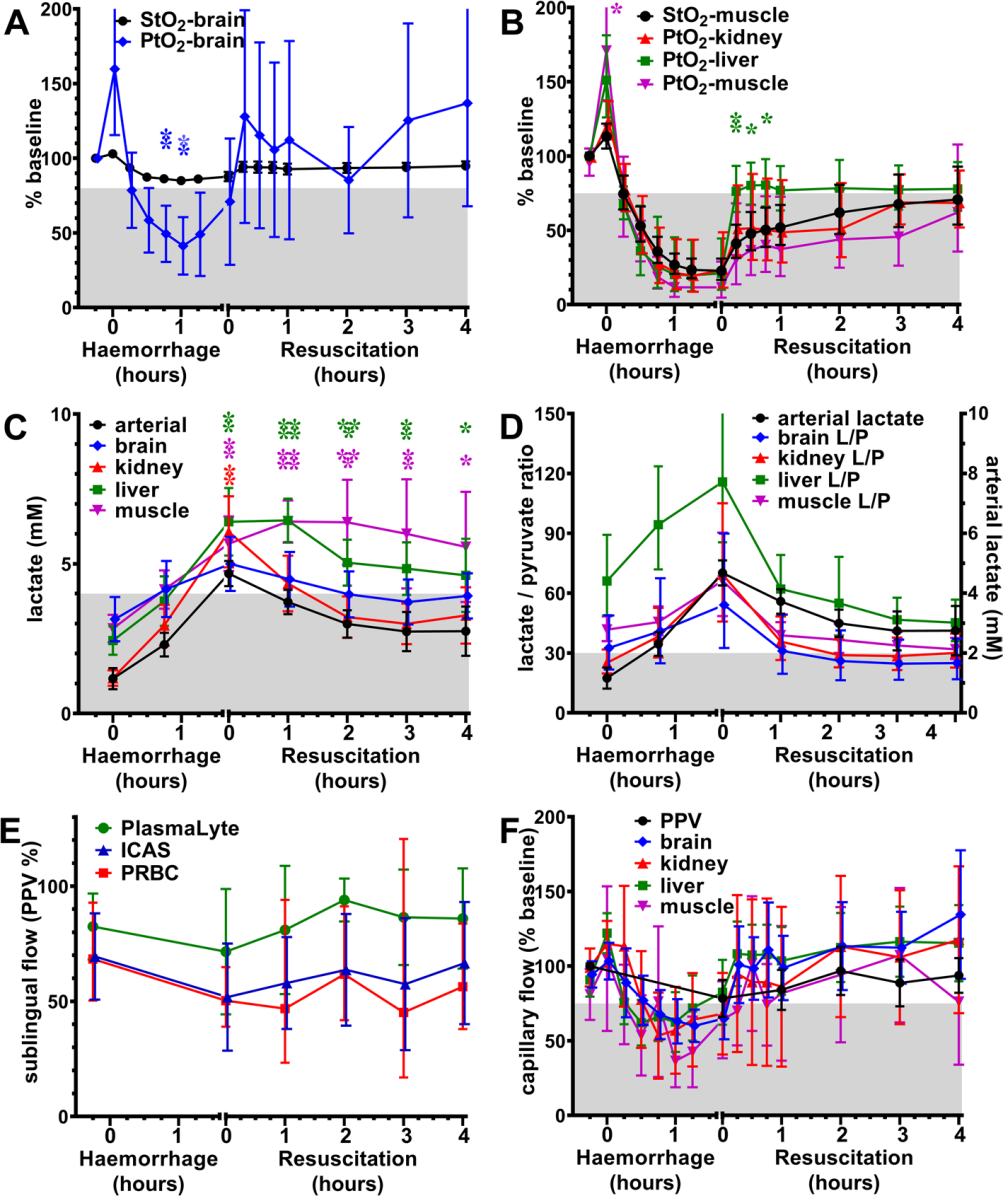
Utility of non-invasive measures to estimate organ-specific outcomes. **A** Concurrent recovery of PtO_2_ and StO_2_ in brain. **B** Liver PtO_2_ recovered (> 75% baseline) before other tissues and muscle StO_2_. **C** Arterial lactate was associated with brain and kidney lactate but delayed liver and muscle lactate clearance (< 4 mM; baseline mean + SD). **D** Arterial lactate was associated with recovery in organ-specific lactate/pyruvate ratios (< 30). **E** Sublingual capillary flow (proportion perfused vessels; PPV) recovered similarly between treatment groups; (**F**) and an increase in sublingual capillary flow indicated that organ-specific capillary flow had improved (> 75% baseline). Data shown as mean or geometric mean according to normality test, with 95% confidence intervals. Mixed model ANOVA with Tukey correction for multiple comparisons; *p < 0.05, **p < 0.01, ***p < 0.001, ****p, 0.0001

To determine if non-invasive clinical measures were reliable surrogates for invasive organ-specific measures, data from all animals were combined and normalised to % baseline for correlations and mixed effects models (Fig. 5). Brain StO_2_ increased when brain PtO_2_ increased (Fig. 5A). Muscle StO_2_ mirrored internal organ PtO_2_ during haemorrhage, but liver PtO_2_ recovered earlier than muscle StO_2_ (*p* = 0.004; Fig. 5B). Combining hourly measures during recovery, brain StO_2_ correlated with brain PtO_2_ (*r* = 0.257; *p* = 0.0048; *n* = 119), and muscle StO_2_ correlated with muscle PtO_2_ (*r* = 0.4036; *p* < 0.0001; *n* = 112), but not with kidney or liver PtO_2_ which recovered earlier. Clearance of arterial lactate correlated with lactate in brain (*r* = 0.356; *p* < 0.0001; *n* = 119), kidney (*r* = 0.790; *p*, 0.0001; *n* = 119), liver (*r* = 0.754; *p* < 0.0001; *n* = 118) and muscle (*r* = 0.583; *p* < 0.0001; *n* = 118), but lactate remained elevated in liver and muscle (*p* < 0.01) compared to arterial lactate (Fig. 5C). Lactate/pyruvate ratios < 30 define metabolic recovery, and improvements tracked with arterial lactate (Fig. 5D). Sublingual capillary flow (PPV) recovered similarly between groups (Fig. 5E). Organ-specific capillary flow tended to increase more rapidly than sublingual flow during the first hour (Fig. 5F), but both measurements confirmed maximal recovery of capillary flow at 2 h treatment. If measured earlier, PPV nadir may also have occurred at 60 min haemorrhage, as measured by Doppler probes in each organ.

### Treatment effects on inflammation and organ function

**Fig. 6:**
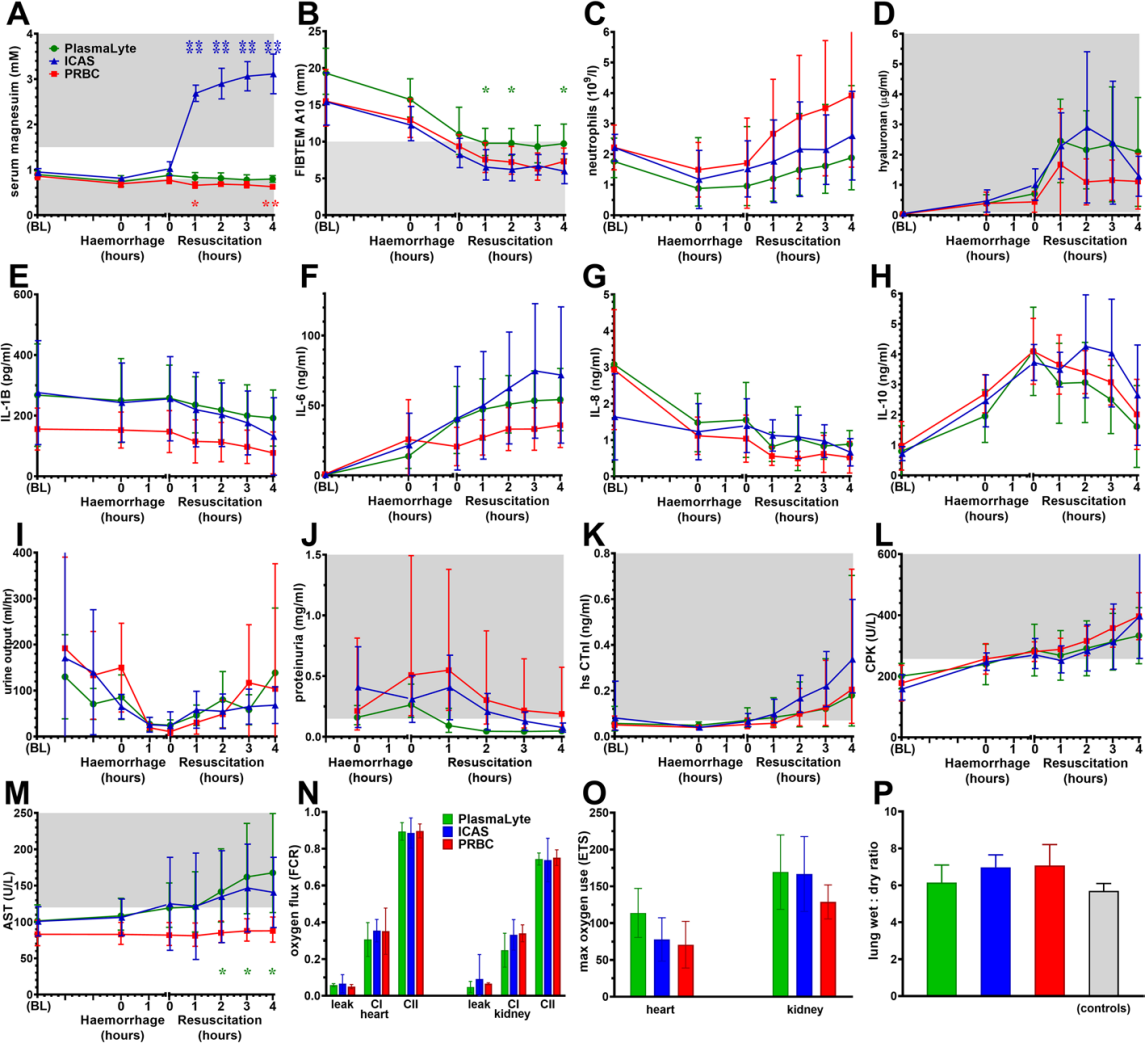
Treatment effects on inflammation and organ function. **A** Serum magnesium. **B** 10 min ROTEM amplitude (A10) in FIBTEM. **C** Circulating neutrophil count. **D** Serum hyaluronan; inflammatory cytokines in plasma (**E**) IL-1β, (**F**) IL-6, (**G**) IL-8, and (**H**) IL-10. **I** Urinary output. **J** Proteinuria. **K** Serum cardiac troponin-I. **L** Serum creatine phosphokinase. **M** Serum aspartate aminotransferase. **N** Cardiac and renal tissue mitochondrial oxygen consumption (FCR: flux-controlled ratio), background vs. complex I and II. **O** Total mitochondrial electron transfer capacity; and (**P**) lung wet/dry ratios. Data shown as mean or geometric mean according to normality test, with 95% confidence intervals. Mixed model ANOVA with Tukey correction for multiple comparisons; *p < 0.05, **p < 0.01, ***p < 0.001, ****p, 0.0001

We assessed effects of treatment on haematological, inflammatory and tissue/organ function markers (Fig. 6). ICAS increased serum magnesium levels (*p* < 0.0001) into mild hypermagnesemia associated with increased peripheral vasodilation (Fig. 6A). Fibrinogen levels decreased during instrumentation, haemorrhage and fluid treatment, but remained higher (*p* < 0.05) in PlasmaLyte-treated animals (Fig. 6B). PRBC tended to increase circulating neutrophils in more animals compared to crystalloid treatment (Fig. 6C). Plasma hyaluronan levels tended to increase more during crystalloid treatment (Fig. 6D). The inflammatory cytokine response was similar between groups (Fig. 6E–H). IL-1β and IL-8 declined throughout the procedure. IL-6 and IL-10 increased during surgical instrumentation and haemorrhage. However, IL-6 tended to increase more during crystalloid treatment. After 3 h treatment, IL-6 levels correlated positively with average fluid rates (Spearman *r* = 0.506; *p* = 0.012), which tended to be higher in crystalloid-treated animals. Urinary output and proteinuria recovered similarly between groups (Fig. 6I, J), whereas cardiac troponin-I remained elevated in all groups (Fig. 6K). Creatine phosphokinase increased in all groups (Fig. 6L), while aspartate aminotransferase increased (*p* < 0.05) after crystalloid treatment (Fig. 6M). Increased creatine phosphokinase and aspartate aminotransferase were associated with muscle injury and not liver, because the liver-specific enzymes gamma-glutamyl transpeptidase declined and alkaline phosphatase remained at normal levels (Additional file: 1: Fig. S1). High-resolution respirometry demonstrated no significant difference in respiratory capacities at any level of the mitochondrial electron transfer system in heart or kidney (Fig. 6N, O). Post-mortem lung wet/dry ratios (Fig. 6P) were similar between groups, and comparable to published data from control anaesthetised sheep [38], which demonstrated that fluid resuscitation did not significantly increase lung oedema after haemorrhagic shock.

## Discussion

The principal findings of this study using a sheep model were (i) no apparent differences in outcomes when PRBC or two different crystalloid solutions were used to treat severe haemorrhagic shock, (ii) non-invasive technologies appear appropriate for estimating vital organ oxygen delivery and metabolism, and (iii) recovery of tissue oxygen delivery after substantial haemodilution to below 70 g/L, confirmed that restrictive transfusion thresholds are appropriate, and may be advantageous. The novel crystalloid ICAS exhibited vasodilatory activity and tended to reduce time to achieve the primary composite oxygen delivery outcome. Reliable estimation of oxygen delivery to vital tissues may augment Patient Blood Management protocols to accommodate reduced haemoglobin thresholds where possible. Adaptation of this model to explore tissue oxygen delivery under increasing haemodilution may reveal the organ-specific, haemodynamic and physiologic parameters that define “critical haemoglobin”, reportedly ≤ 50 g/L in most acute haemorrhage scenarios [17, 39, 40].

PRBC transfusion did not significantly reduce the time to achieve the composite haemodynamic outcome compared to crystalloid treatment. In agreement with other studies [41], we observed non-significant trends toward lower CI and higher SVRI after PRBC compared to fluid treatment, but PRBC transfusion did not significantly increase MAP nor decrease HR compared to crystalloid treatment. The inability to recover MAP to baseline levels in all treatment arms was likely due to plasma protein dilution and reduced oncotic pressure [42]. Reduced fibrinogen levels in all animals may have contributed to increased plasma hyaluronan from glycocalyx breakdown [43], as frequently observed in traumatic haemorrhagic shock [44]. Non-haemoglobin interventions to increase plasma viscosity after haemodilution may improve renal perfusion [45], in the same way that increased haematocrit-associated blood viscosity improves tissue perfusion [46]. Therefore, appropriate Patient Blood Management is not necessarily haemoglobin-dependent.

Despite trends for ICAS to increase peripheral vasodilation and fluid requirements, equivalent outcomes were observed for lung wet/dry ratio, endothelial glycocalyx integrity, renal and cardiac function, mitochondrial function, inflammatory cytokine levels, and other tissue function markers. ICAS has a physiological mineral composition similar to plasma, except vasoactive levels of magnesium, and includes trace concentrations of nitrate, nitrite, metals and metalloids [27]. Early 1900’s canine haemorrhagic shock experiments demonstrated increased post-resuscitation vitality and survival with this fluid compared to saline [47]. Recently, a porcine haemorrhagic shock model demonstrated equivalent haemodynamic, oxygen delivery and survival outcomes with this fluid compared to whole blood transfusion [27]. In our ovine model, outcomes from ICAS treatment were also comparable to PRBC with Hartmann’s. During the first treatment hour, peripheral muscle perfusion and PtO_2_ recovered earlier after ICAS, but renal perfusion and PtO_2_ recovered earlier after PRBC, but these differences were insignificant thereafter. Although lactate levels were similar between groups, more animals treated with ICAS remained in base deficit, due to lack of bicarbonate equivalents compared to PlasmaLyte and Hartmann’s. These combined observations suggest an optimised treatment for haemorrhagic shock could include a mineral-diverse fluid such as ICAS but with less magnesium to moderate peripheral vasodilation, supplemented with bicarbonate equivalents, and used with a colloid to increase MAP and fibrinogen to maintain haemostasis and glycocalyx integrity.

We demonstrated utility of non-invasive point-of-care measures of tissue microvascular perfusion and oxygen delivery as reliable surrogates of vital organ status. Functional capillary density is closely associated with tissue oxygen delivery and organ survival [2, 19, 48, 49]. Our data confirmed that recovery of sublingual capillary perfusion mirrored recovery of vital organ perfusion; also confirmed for renal perfusion in another haemodilution study [45]. Arterial lactate was a reliable non-invasive surrogate of lactate clearance from vital organs. NIRS measures regional tissue oxygen delivery as oxyhaemoglobin saturation in all blood vessels, including venous blood which remains relatively desaturated until tissue oxygen supply is adequately reinstated. Laser-optic probe assessment of interstitial dissolved oxygen (PtO_2_) represents the local balance between oxygen supply from perfused capillaries and oxygen consumption in cells. Hepatic and renal PtO_2_ recovered earlier than muscle StO_2_ but brain PtO_2_ and StO_2_ recovered concurrently. Therefore, improved StO_2_ may predict adequate vital organ oxygen delivery. However, reliability of NIRS in not universal. First-gen NIRS platforms were not considered acceptable for cerebral assessment because of interference from extracranial blood [50]. The next-gen NIRS platform used in our study controlled for extracranial saturation using five wavelengths with deep and shallow tissue sensors [51], and cerebral cortex and peripheral muscle oxygen re-saturation occurred independently. Furthermore, theoretical signal contamination between cerebral and skin oxygen saturation during shock is trivial when peripheral tissue saturation is low; a conclusion supported by hypoxia studies using next-gen NIRS platforms [52]. Innovative point-of-care technologies worthy of appraisal for Patient Blood Management include broadband NIRS which measures tissue oxygen saturation and metabolite levels [53], and transdermal respirometry for real-time mitochondrial oxygen tension [54].

Limitations of this animal model of haemorrhagic shock and species-specific response to resuscitation were outlined previously [28]. We could not confirm the long-term impact of fluid dosing on organ function outcomes, which was not feasible with this highly invasive protocol. Early restrictive fluid dosing in trauma is associated with reduced inflammation and improved organ and survival outcomes [3, 4], while goal-directed fluid dosing may benefit both surgical and trauma settings [55, 56]. Our haemorrhagic shock model was designed for controlled perioperative bleeding and fluid management. Although we used goal-directed fluid dosing targeting MAP > 65 mmHg, high fluid rates correlated with inflammatory IL-6 levels. Therefore, in the absence of extended survival analysis, our observations of tissue oxygen delivery and short-term organ function outcomes cannot be extrapolated to organ survival in clinical practice.

Other technical limitations of our model included the effect of invasive instrumentation on baseline inflammation, although this is also observed in surgical and trauma patients. We could not determine the proximity of invasive Doppler probe tips to larger blood vessels, which may have contributed to high variability in organ-specific microvascular flow data, although overall trends were credible. Management of FiO_2_ and other ventilatory parameters was representative of ICU care, but less relevant in pre-hospital scenarios. Randomisation was largely effective, but did not eliminate tendency to low P/F ratios in PlasmaLyte-treated animals and higher baseline kidney PtO_2_ in PRBC-treated animals. Haemodynamic response to haemorrhage, particularly tachycardia, was not evenly distributed among groups, which reflects real-world variation in patients presenting with haemorrhagic shock. Notwithstanding cost and ethical considerations, an increased number of animals may not have overcome the inherent variability observed in large animal models.

## Conclusions

Blood volume restitution restored haemodynamic parameters and tissue oxygen delivery, whether haemoglobin was maintained at normal levels after PRBC transfusion, or haemodiluted to below what is considered a restrictive transfusion threshold after fluid resuscitation. Non-invasive sublingual microvascular imaging, NIRS, and blood lactate, are promising point-of-care technologies that could be used to predict microvascular blood flow, tissue oxygen delivery and metabolic recovery in vital organs. These physiological measures could be used to guide Patient Blood Management-associated treatments for massive haemorrhage, and determine whether transfusion is warranted when haemoglobin levels fall below 70 g/L. Further investigation of novel vasoactive fluids such as ICAS for haemorrhagic shock is warranted, along with clinical studies to assess the role and impact of NIRS and sublingual microvascular imaging in the management of acute haemorrhage.

## Supplementary Information

**Additional file 1**: Method: high-resolution respirometry, and Figure S1: serum GGT and ALT.

## Data Availability

The data sets used and/or analysed during the current study are available from the corresponding author on reasonable request.

## Abbreviations

PRBC: Packed red blood cells
ICAS: Isotonic crystalloid aqueous solution
NIRS: Near infra-red spectroscopy
MAP: Mean arterial pressure
HR: Heart rate
CI: Cardiac index
SVRI: Systemic vascular resistance index
StO2: Regional tissue oxygen saturation
SvO2: Mixed venous saturation
PtO2: Tissue oxygen tension

## References

[1] Lozano R, Naghavi M, Foreman K (2012). Global and regional mortality from 235 causes of death for 20 age groups in 1990 and 2010: a systematic analysis for the Global Burden of Disease Study 2010. Lancet.

[2] Siegemund M, Hollinger A, Gebhard EC (2019). The value of volume substitution in patients with septic and haemorrhagic shock with respect to the microcirculation. Swiss Med Wkly.

[3] Jiang LM, He J, Xi XY (2019). Effect of early restrictive fluid resuscitation on inflammatory and immune factors in patients with severe pelvic fracture. Chin J Traumatol.

[4] Tran A, Yates J, Lau A (2018). Permissive hypotension versus conventional resuscitation strategies in adult trauma patients with hemorrhagic shock: a systematic review and meta-analysis of randomized controlled trials. J Trauma Acute Care Surg.

[5] Moreno DH, Cacione DG, Baptista-Silva JC (2018). Controlled hypotension versus normotensive resuscitation strategy for people with ruptured abdominal aortic aneurysm. Cochrane Database Syst Rev.

[6] Stein P, Kaserer A, Sprengel K (2017). Change of transfusion and treatment paradigm in major trauma patients. Anaesthesia.

[7] Winearls J, Campbell D, Hurn C (2017). Fibrinogen in traumatic haemorrhage: a narrative review. Injury.

[8] van Turenhout EC, Bossers SM, Loer SA (2020). Pre-hospital transfusion of red blood cells. Part 2: A systematic review of treatment effects on outcomes. Transfus Med.

[9] Cantle PM, Cotton BA (2017). Balanced resuscitation in trauma management. Surg Clin North Am.

[10] Shea SM, Staudt AM, Thomas KA (2020). The use of low-titer group O whole blood is independently associated with improved survival compared to component therapy in adults with severe traumatic hemorrhage. Transfusion.

[11] Rehn M, Weaver AE, Eshelby S (2018). Pre-hospital transfusion of red blood cells in civilian trauma patients. Transfus Med.

[12] Napolitano LM, Kurek S, Luchette FA (2009). Clinical practice guideline: red blood cell transfusion in adult trauma and critical care. Crit Care Med.

[13] Derzon JH, Clarke N, Alford A (2019). Restrictive transfusion strategy and clinical decision support practices for reducing RBC transfusion overuse. Am J Clin Pathol.

[14] National Blood Authority. Patient Blood Management Guidelines: Module 2 Perioperative. Canberra, ACT, Australia.2012. Available from: https://www.blood.gov.au/pbm-module-2. Accessed Sept 2021

[15] Whitlock EL, Kim H, Auerbach AD (2015). Harms associated with single unit perioperative transfusion: retrospective population based analysis. BMJ.

[16] Shander A, Javidroozi M, Ozawa S (2011). What is really dangerous: anaemia or transfusion?. Br J Anaesth.

[17] Salpeter SR, Buckley JS, Chatterjee S (2014). Impact of more restrictive blood transfusion strategies on clinical outcomes: a meta-analysis and systematic review. Am J Med.

[18] Scheuzger J, Zehnder A, Meier V (2020). Sublingual microcirculation does not reflect red blood cell transfusion thresholds in the intensive care unit-a prospective observational study in the intensive care unit. Crit Care.

[19] Tsai AG, Friesenecker B, Intaglietta M (1995). Capillary flow impairment and functional capillary density. Int J Microcirc Clin Exp.

[20] Benni PB, MacLeod D, Ikeda K (2018). A validation method for near-infrared spectroscopy based tissue oximeters for cerebral and somatic tissue oxygen saturation measurements. J Clin Monit Comput.

[21] Bjerkvig CK, Strandenes G, Eliassen HS (2016). "Blood failure" time to view blood as an organ: how oxygen debt contributes to blood failure and its implications for remote damage control resuscitation. Transfusion.

[22] Spahn DR, Bouillon B, Cerny V (2019). The European guideline on management of major bleeding and coagulopathy following trauma: fifth edition. Crit Care.

[23] Cole E, Weaver A, Gall L (2019). A decade of damage control resuscitation: new transfusion practice, new survivors, new directions. Ann Surg.

[24] Goobie SM, Shander A (2020). One size does not fit all in treating massive hemorrhage. Anesth Analg.

[25] Weinberg L, Collins N, Van Mourik K (2016). Plasma-Lyte 148: a clinical review. World J Crit Care Med.

[26] Oller Duque L, Shander A (2018) Isotonic Crystalloid Aqueous Solution. World Intellectual Property Organization. WO/2018/019663

[27] Oller L, Dyer WB, Santamaría L (2019). The effect of a novel intravenous fluid (Oxsealife^®^) on recovery from haemorrhagic shock in pigs. Anaesthesia.

[28] Dyer WB, Tung JP, Li Bassi G (2021). An ovine model of haemorrhagic shock and resuscitation, to assess recovery of tissue oxygen delivery and oxygen debt, and inform Patient Blood Management. Shock.

[29] Perry M (1998). Revised Australian code of practice for the care and use of animals for scientific purposes. Aust Vet J.

[30] Simonova G, Tung JP, Fraser JF (2014). A comprehensive ovine model of blood transfusion. Vox Sang.

[31] Chemonges S, Shekar K, Tung JP (2014). Optimal management of the critically ill: anaesthesia, monitoring, data capture, and point-of-care technological practices in ovine models of critical care. Biomed Res Int.

[32] Wilson DV, Evans AT, Carpenter RA (2004). The effect of four anesthetic protocols on splenic size in dogs. Vet Anaesth Analg.

[33] Musk GC, Kershaw H, Kemp MW (2019). Anaemia and hypoproteinaemia in pregnant sheep during anaesthesia. Animals.

[34] Dooley PC, Hecker JF, Webster ME (1972). Contraction of the sheep's spleen. Aust J Exp Biol Med Sci.

[35] Hodgetts VE (1961). The dynamic red cell storage function of the spleen in sheep. III. Relationship to determination of blood volume, total red cell volume, and plasma volume. Aust J Exp Biol Med Sci.

[36] Foley SR, Solano C, Simonova G (2014). A comprehensive study of ovine haemostasis to assess suitability to model human coagulation. Thromb Res.

[37] Bouquet M, Passmore MR, See Hoe LE (2020). Development and validation of ELISAs for the quantitation of interleukin (IL)-1β, IL-6, IL-8 and IL-10 in ovine plasma. J Immunol Methods.

[38] Julien M, Flick MR, Hoeffel JM (1984). Accurate reference measurement for postmortem lung water. J Appl Physiol Respir Environ Exerc Physiol.

[39] Shander A, Javidroozi M, Naqvi S (2014). An update on mortality and morbidity in patients with very low postoperative hemoglobin levels who decline blood transfusion (CME). Transfusion.

[40] Hong T, Shander A, Agarwal S (2015). Management of a Jehovah's witness patient with sepsis and profuse bleeding after emergency coronary artery bypass graft surgery: rethinking the critical threshold of oxygen delivery. A A Case Rep.

[41] Saugel B, Klein M, Hapfelmeier A (2013). Effects of red blood cell transfusion on hemodynamic parameters: a prospective study in intensive care unit patients. Scand J Trauma Resusc Emerg Med.

[42] Manning RD, Guyton AC (1983). Effects of hypoproteinemia on fluid volumes and arterial pressure. Am J Physiol.

[43] Wu F, Chipman A, Pati S (2020). Resuscitative strategies to modulate the endotheliopathy of trauma: from cell to patient. Shock.

[44] Halbgebauer R, Braun CK, Denk S (2018). Hemorrhagic shock drives glycocalyx, barrier and organ dysfunction early after polytrauma. J Crit Care.

[45] Ergin B, van Rooij T, Lima A (2021). Hydroxyl Ethyl Starch (HES) preserves intrarenal microcirculatory perfusion shown by contrast-enhanced ultrasound (CEUS), and renal function in a severe hemodilution model in pigs. Shock.

[46] Cabrales P, Intaglietta M, Tsai AG (2007). Transfusion restores blood viscosity and reinstates microvascular conditions from hemorrhagic shock independent of oxygen carrying capacity. Resuscitation.

[47] Quinton R (1912) L'eau de Mer; Meileu Organique. Editor: Masson. https://openlibrary.org/books/OL24240755M/L%27eau_de_mer_milieu_organique. Accessed 5 Nov 2021

[48] Tsai AG, Vazquez BY, Hofmann A (2015). Supra-plasma expanders: the future of treating blood loss and anemia without red cell transfusions?. J Infus Nurs.

[49] Villela NR, Salazar Vazquez BY, Intaglietta M (2009). Microcirculatory effects of intravenous fluids in critical illness: plasma expansion beyond crystalloids and colloids. Curr Opin Anaesthesiol.

[50] Davie SN, Grocott HP (2012). Impact of extracranial contamination on regional cerebral oxygen saturation: a comparison of three cerebral oximetry technologies. Anesthesiology.

[51] Greenberg S, Murphy G, Shear T (2016). Extracranial contamination in the INVOS 5100C versus the FORE-SIGHT ELITE cerebral oximeter: a prospective observational crossover study in volunteers. Can J Anaesth.

[52] Dixon B, MacLeod DB (2020). Assessment of a non invasive brain oximeter in volunteers undergoing acute hypoxia. Med Devices.

[53] Lange F, Bale G, Kaynezhad P (2020). Broadband NIRS cerebral evaluation of the hemodynamic and oxidative state of cytochrome-c-oxidase responses to +Gz acceleration in healthy volunteers. Adv Exp Med Biol.

[54] Mik EG, Balestra GM, Harms FA (2020). Monitoring mitochondrial PO2: the next step. Curr Opin Crit Care.

[55] Messina A, Robba C, Calabrò L (2021). Association between perioperative fluid administration and postoperative outcomes: a 20-year systematic review and a meta-analysis of randomized goal-directed trials in major visceral/noncardiac surgery. Crit Care.

[56] Ramesh GH, Uma JC, Farhath S (2019). Fluid resuscitation in trauma: what are the best strategies and fluids?. Int J Emerg Med.

