# Supplementary material for: Recovery of organ-specific tissue oxygen delivery at restrictive transfusion thresholds after fluid treatment in ovine haemorrhagic shock
Source: Intensive Care Med Exp. 2022 Apr 4;10:12. doi: 10.1186/s40635-022-00439-6 (PMC8980119; doi:10.1186/s40635-022-00439-6)
Supplement: Supplementary file 1 — Additional file 1: Method: high-resolution respirometry, and Figure S1: serum GGT and ALT. [file 40635_2022_439_MOESM1_ESM.pdf]

Additional file 1 – Method and Figure S1

Method: high resolution respirometry (HRR)

Mitochondrial respiration was measure in homogenised tissue of the right ventricle and renal cortex using a high-resolution respirometer Oroboros® O2k-Oxygraph (Oroboros Instruments, Innsbruck, Austria) at 37°C. Immediately after euthanasia, using a needle biopsy, three myocardial biopsies were taken at the base, mid and apical regions of the right ventricle (10mg/biopsy) as well as three kidney biopsies taken from the superior, middle, and inferior pole of the renal cortex region (10mg/biopsy). For each tissue type, the biopsies were pooled together to mitigate regional variations and obtain a more accurate representation of mitochondrial respiration. Samples were homogenised in 500μL cold respiration buffer MiR05-kit (20mg/mL) using the PBI-Shredder SG3 (Oroboros Instruments, Innsbruck, Austria). To yield a 1mg/mL protein concentration, 50μL of heart or kidney homogenate was injected into each respiratory chamber. Oxygen concentration and flux were simultaneously recorded and analysed by DatLab software (Version 7.4, Oroboros Instruments, Innsbruck, Austria). Before all experiments were commenced, calibration of the respirometer was performed at air saturation, 37 °C. Manual titration of inhibitors and uncouplers in presence of carbohydrates was performed using Hamilton syringes. In accordance with the Oroboros guidelines, the resulting O_2 flux values were utilized to calculate respiration in different coupling control states, including LEAK, complex I and II oxidative phosphorylation and maximum electron transport system (ETS). The substrate-uncoupler-inhibitor titration sequence used for each homogenate was as follows: 5mM pyruvate, 2mM malate, 10mM glutamate, 2mM ADP, 10μM cytochrome c, 10mM succinate, 2mM ADP, 2μM carbonyl cyanide m-chloro phenyl hydrazone (CCCP), 0.5μM rotenone and 2.5μM antimycin A, 2mM ascorbate, 0.5mM TMPD and 100mM azide. LEAK respiration was measured after the addition of reducing substrates (pyruvate, malate, and glutamate) providing NADH to CI. The first addition of ADP allowed to selectively quantify the activity of complex I (CI OXPHOS). 10μM cytochrome c was added to survey the integrity of the outer mitochondrial membrane and CII OXPHOS was measured after succinate and the second ADP addition. Subsequent injections of the uncoupler CCCP allowed to obtain the maximum respiratory activity (ETS) in the uncoupled state and the residual oxygen consumption (ROX) was measured after the addition of antimycin when CI and CII are inhibited. ROX is a respiration connected to other cellular oxygen-consuming processes besides the respiratory chain. Lowest oxygen consumption at ROX was subtracted from LEAK, CI and CII OXPHOS measures and were then corrected for maximum oxygen consumption during ETS. Correcting for maximum ETS capacity is referred to as a flux control ratio (FCR) and mitigates variation due to changes in mitochondrial density or morphology.

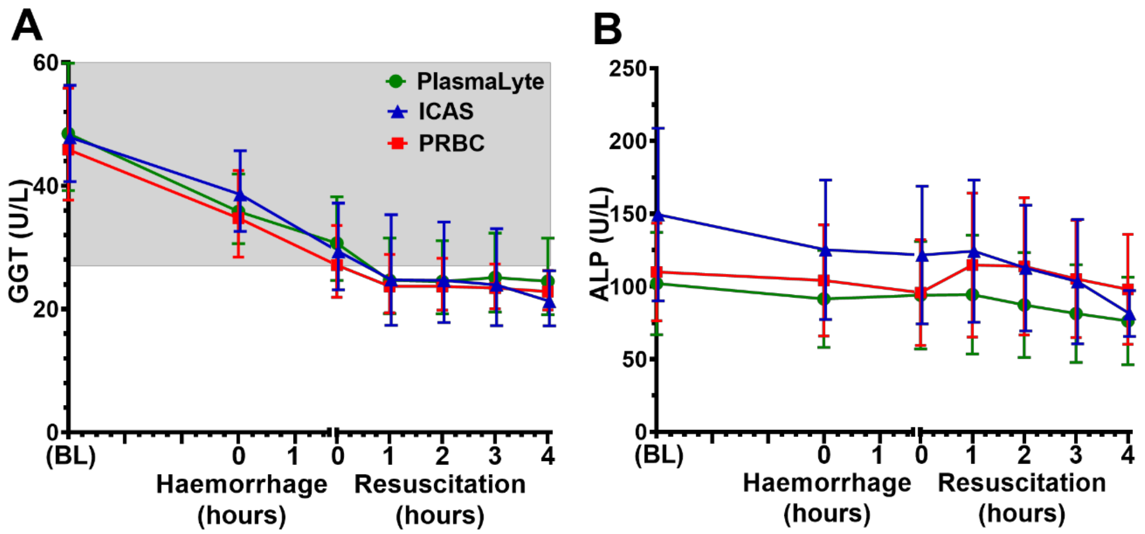

Figure S1. (A) serum gamma-glutamyl transpeptidase, and (B) serum alkaline phosphatase.
